# A Comparison of Hotel Guest Experience Before and During Pandemic: Evidence from Online Reviews

**DOI:** 10.1007/978-3-030-65785-7_52

**Published:** 2020-11-28

**Authors:** Irene Cheng Chu Chan, Jing Ma, Huiyue Ye, Rob Law

**Affiliations:** 1 Department for Informatics, Technical University of Munich, Garching bei München, Bayern Germany; 2 Smart Tourism Education Platform (STEP) College of Hotel and Tourism Management, Kyung Hee University, Seoul, Korea (Republic of); 3 Department of Tourism and Service Management, MODUL University Vienna, Vienna, Wien Austria; 4 School of Hospitality Management, Macao Institute for Tourism Studies, Macao S.A.R., China; 5 School of Hotel and Tourism Management, The Hong Kong Polytechnic University, Hong Kong S.A.R, China

**Keywords:** COVID-19, Pandemic, Guest experiences, Hotel, Online reviews

## Abstract

This paper compares the determinants of guest experience at luxury hotels in Mainland China before and during the pandemic—COVID-19. In particular, 740 Chinese reviews posted before the pandemic outbreak, and 1283 reviews posted during the pandemic were collected. Text analytics were applied to segment and count the frequency of words in these online reviews. The results show that the core dimensions of guest experiences at luxury hotels include services, room quality and settings, hotel facilities, dining, location, and environment. These core dimensions do not change regardless of the period before or during the pandemic. However, guests have higher expectations on hotel services such as late check-out and delivery service of takeaway during the pandemic. Online reviews amid-pandemic also contain words related to pandemic prevention and control measures, such as guest traffic and body temperature. Suggestions on operations and management are provided for hotel practitioners to improve their services during the critical period.

## Introduction

Most, if not all, industries have been facing unprecedented challenges since the outbreak of the Novel Coronavirus (COVID-19), and tourism has been the hardest hit among all [11]. The United Nations World Tourism Organization [20] estimated a 98% drop in international tourist arrivals in May 2020. According to STR [19], China has experienced a slump in tourism demand and a 20.6% decline in domestic tourism revenue in year 2020. Besides, the overall hotel occupancy in China has decreased by 89% from early to mid-January and the overall revenue per available room witnessed a year-on-year drop of over 85%.

Crisis affects the macro-environment and brings about changes in customer behaviors and hotel performances [3, 18]. The outbreak of COVID-19 may have brought about changes in guest experiences at hotels [8, 25]. Customers normally describe their experiences and emotions after their hotel stay in the form of user-generated content, such as online reviews [1]. Therefore, online reviews are frequently used by practitioners to understand the nature and structure of guest experiences, thereby devise useful reactive strategies [20].

Hotels are customer-centric that should keep up with customer preferences and requirements. Thus, it is essential to track the potential changes of guest experiences caused by the impact of COVID-19. In view of that, this study seeks to reveal the differences in guest experiences at luxury hotels before and during the pandemic as reflected in the online reviews posted in those two periods. A text analytic approach is used to transform unstructured data and identify factors in online reviews to compare the determinants of hotel guest experiences before and during the pandemic. The results of this study will offer practical suggestions to hotel managers to develop effective operational and marketing strategies to improve guest experiences at luxury hotels during the challenging situation. Future research opportunities are also provided.

## Literature Review

### Hotel Guest Experience and Online Reviews

Guest experience refers to the cognitive and affective responses derived from the interactions between the guest and a product or service [10, 21]. It is co-produced by service providers and consumers [5]. Xiang, Schwartz, Gerdes Jr, and Uysal [22] identified 80 guest experience-related words, including room, quality, and service. Knutson, Beck, Kim, and Cha further identified the guest experience dimensions by developing a four-factor model of Hotel Experience Index (HEI) [14]. The 18-item index consists of four dimensions, namely environment, accessibility, driving benefit, and incentive [14]. According to Xiang et al. [22], guest experience is used to measure hotel guest satisfaction. Satisfaction with and repurchase of a hotel product may be driven by a set of attributes, such as staff service quality, room quality, amenities, value, and security [6].

With the popularity of sharing experiences through online platforms, online reviews represent a legitimate source for hotel managers to understand customers’ evaluations of hotel service and products [15, 22]. Research on textual reviews in hospitality mainly focus on identifying the hotel attributes and sentiments expressed in the reviews [e.g., 7, 12] and examining the relationships between specific attributes in the textual reviews and the overall review ratings [15, 16]. Comparisons of hotel attributes and guest experiences are mainly done among different hotel guest segments [12] and hotel types [1]. Hong et al. [13] found out that guests placed more emphasis on natural and safe experience associated with the bed & breakfast (B&B) after COVID-19, and provided practical suggestions for the industry to survive the disaster, such as avoid using central air-conditioning and adopting semi-self-service technologies. The current study contributes to existing literature by identifying the changes in hotel guest experience and its determinants before and during a pandemic situation.

## Methodology

### Data Collection

Online reviews were collected from the luxury hotels in Shanghai for two reasons. First, Shanghai is one of the top destinations in Mainland China, with 8.94 million tourist arrivals in 2018 [4]. The large number of visitors fueled the development of the hotel industry in the city [24]. Second, Shanghai has swiftly overcome its semi-lockdown amid the pandemic, unlike the other worst-hit cities, which was under full-lockdown for over one month, such as Wuhan. As a resilient tourism destination, Shanghai shows signs of recovery in March 2020 and has even become the most popular destination in Mainland China after going through the peak of the pandemic in May 2020 [17]. Thus, data before and during the pandemic are more readily available for luxury hotels in Shanghai, considering that travel activities are still present during the pandemic.

Online reviews for 20 luxury hotels in Shanghai that were posted from August 2019 to July 2020 were collected using Google Sheets in August 2020. These 20 hotels were randomly selected based on the list of luxury hotels in Shanghai that is available on Ctrip. Mainland China announced the lockdown measures to control the spread of COVID-19 around the country in late January 2020 [2]. Thus, online reviews posted from August 2019 to January 2020 are categorized into the pre-COVID-19 group (740 reviews), while those posted from February 2020 to July 2019 are in the amid-COVID-19 group (1238 reviews). There is a significant difference in the number of reviews between these two periods. A closer look into the distribution shows that a very small amount of reviews has been posted in September and October 2019 before the outbreak of the pandemic. However, a lot of reviews have been posted in June and July 2020 during the pandemic. This suggests that consumers have a higher motivation to share their experiences with and help the others amid-crisis.

### Data Analysis

The online reviews were analyzed using the Chinese Text Analyzer software, which helps to perform segmentation and frequency count of the words. The authors then reviewed and verified that all retained words are related to guest experiences with hotel, i.e., both the tangible and non-tangible aspects of hotel products [22]. In the first step, frequency analysis using the Chinese Text Analyzer generated a list of words describing hotel guest experiences. In the next step, the primary coder reviewed each word and combine synonyms together (e.g., “writing desk” and “table”, “hygiene”, and “cleanliness”), so as to reduce the number of words. At the same time, words that are irrelevant to hotel guest experiences were eliminated. This iterative coding process seeks to establish a dictionary of terms relevant to hotel guest experiences. The manual coding was done with reference to other similar research [1, 23].

## Findings

### Comparison Between Pre- and Amid-Pandemic

A total of 3,558 and 5,034 words have been segmented from the pre-pandemic and amid-pandemic online reviews respectively. Table 1 shows the proportion of each identified determinants in hotel guest reviews both before and during the pandemic. The results show that the core determinants of hotel guest experience are the same in both periods, and they include services, room quality and settings, hotel facilities, dining, location, and environment. All these six factors have been mentioned more than 10% in the online reviews of the sampled luxury hotels in Shanghai.

**Table 1.** Frequencies of hotel guest experience determinants before and during pandemic

Determinants	Words	% (pre)	% (amid)
Services	Service, check-in, check-out, reception, manager, employee, takeaway, courier, deposit, attitude, waiting, integrity, turn-down	17.3%	21.8%
Room quality and settings	Air-con, power, charging, refrigerator, dressing room, hair dryer, floor, carpet, trash, walls, desk, bed, room, size, design, pillows, fan, smart, heating, windows, clean, appliances, balcony, TV, speaker, mirror, wardrobe	17.7%	16.1%
Hotel facilities	Spa, parking, fitness, children’s playground, facilities, entrance, public toilets, lobby, event, signs, show, meeting rooms, billiard, sauna, steam, robots, waterpark, activities, swimming pool, lawn, greening, garden, internet speed, shuttle, elevator, singers	14.8%	13.9%
Dining	Café, executive lounge, bar, drinks, restaurant, meals, food	10.9%	11.6%
Location	Transportation, location surroundings, nearby attractions, peripheral facilities, surrounding scenery, convenient, beaches, lake, road, farm	12.5%	10.9%
Environment	Humidity, noise, quiet, atmosphere, environment, smell, mosquitoes, soundproofing, scent, view	10.4%	10.3%
Value/price	Price, discount, upgrade, value for money	6.7%	5.8%
Health measures	Guest traffic, COVID-19, body temperature, pandemic safety, impact of the pandemic, pandemic prevention and control, isolated hotel, carry-on code, central isolation	0.8%	3.2%
Bathroom	Drainage, clothes hanger, water, bathing, washing, bathrooms, towel, shower, toilet	3.3%	2.5%
Amenities	Disposable supplies, ice, coffee machines, gifts, slippers, fruit, bath products, toothbrush and toothpaste, birthday, anti-mosquito, drinking water, tableware, mask	2.8%	2.4%
Hotel design	Hotel size, feel, style, color, hotel decoration	2.7%	0.4%
Management	Membership, member discount, management, policy	0.1%	0.3%
Safety & privacy	Privacy, safety, security, risk	0.1%	0.2%
Maintenance	Works, repairs, maintenance	0%	0.1%

The proportions for these factors are similar in the pre- and amid-pandemic periods, except for services. Hotel guests mentioned much more about services during the pandemic (21.8%) compared to the time before (17.3%). A closer look at the review content shows that the significant rise in the proportion of reviews describing the service dimension is mainly due to two items, including check-out time and takeaway services. A few reviews mentioned that the hotel actively offered late check-out during the pandemic. For example, “*The hotel management is very nice, they extended the check*-*out time for us without extra charges. Superb*!” On the other hand, some guests stated that the hotel does not help with delivering the takeaway that they have ordered. For example, one guest mentioned *“Overall the hotel is nice, the facilities are smart, but they refused to help send the take*-*away up to the room. They should improve on this service.”*

As anticipated, the proportion of reviews mentioning about health measures increases from 0.8% before the outbreak of the pandemic to 3.2% during the pandemic. Words that most frequently appeared in the online reviews related to this dimension include *guest traffic* (59 times), *pandemic prevention and control* (46 times), and the *impact of pandemic* (24 times).

## Conclusions

### Implications

The findings of this study reveal that most dimensions of guest experiences at luxury hotels, including room quality and settings, hotel facilities, location, environment, value/price, bathroom, amenities, and hotel design, show a minuscule decrease in frequencies, whereas the remaining determinants, conversely, show an increase. What is worth noticing is that “services” show an increase in proportions, indicating that hotel guests expect to receive additional services, such as delivery service of takeaway and late check-out services amid-pandemic. Besides, guests become more concerned about whether the hotel is taking preventive measures and control during the pandemic, such as controlling the number of guests at the hotel, measuring body temperature. This can be explained by the fact that people are more concerned about public health and personal safety during the pandemic. Social distancing prevents the virus from further spreading [9]. While it is important for hotels to maintain their standard quality of products and services as usual, hotel managers may provide more proactive or even extra services to guests during this critical period. As reflected in this study, offering late check-out and assisting with the delivery of takeaways may maintain customers’ perceived service quality of the hotel. In order to reduce interactions between service employees and guests, robots may be used to take and delivery takeaways and room services to guestrooms. Furthermore, self-service kiosk and express check out services may be offered to reduce the waiting time of guests, thereby avoid gathering large crowds at the hotel lobby. Any measures taken by the hotel in preventing the pandemic should be carefully communicated with hotel guests on the hotel websites or during the booking stage, so as not to create any inconveniences during the service experience.

### Limitations and Future Research

The findings of this study are based on descriptive analysis of online reviews from 20 luxury hotels in Shanghai. The focus on luxury hotels and small samples size may limit the generalizability of the findings. Besides, ratings and sentiments in the online reviews have not been considered. Nevertheless, this study sheds lights on the potential differences in hotel guest experiences caused by the outbreak of the pandemic. Future studies may build on the idea of this study and expand the scope of the study to include all luxury hotels, or even all hotels across different categories in Shanghai and other cities of China. The dictionary developed in this study may be used for machine learning so that a larger amount of reviews may be analyzed. Analysis on the ratings and sentiments in the review text may be performed to reveal further insights into the differences in guests’ sentiments before and during the pandemic.
